# *Pneumocystis jirovecii—*from a commensal to pathogen: clinical and diagnostic review

**DOI:** 10.1007/s00436-015-4678-6

**Published:** 2015-08-19

**Authors:** Magdalena Sokulska, Marta Kicia, Maria Wesołowska, Andrzej B. Hendrich

**Affiliations:** Department of Biology and Medical Parasitology, Wrocław Medical University, ul. Mikulicza-Radeckiego 9, 50-367 Wrocław, Poland

**Keywords:** *Pneumocystis jirovecii*, *Pneumocystis* pneumonia, Opportunistic infection, Colonization

## Abstract

*Pneumocystis* pneumonia is an opportunistic disease caused by invasion of unicellular fungus *Pneumocystis jirovecii.* Initially, it was responsible for majority of morbidity and mortality cases among HIV-infected patients, which later have been reduced due to the introduction of anti-retroviral therapy, as well as anti-*Pneumocystis* prophylaxis among these patients. *Pneumocystis* pneumonia, however, is still a significant cause of mortality among HIV-negative patients being under immunosuppression caused by different factors, such as transplant recipients as well as oncologically treated ones. The issue of pneumocystosis among these people is particularly emphasized in the article, since rapid onset and fast progression of severe symptoms result in high mortality rate among these patients, who thereby represent the group of highest risk of developing *Pneumocystis* pneumonia. In contrast, fungal invasion in immunocompetent people usually leads to asymptomatic colonization, which frequent incidence among healthy infants has even suggested the possibility of its association with sudden unexpected infant death syndrome. In the face of emerging strains with different epidemiological profiles resulting from genetic diversity, including drug-resistant genotypes, the colonization phenomenon desires particular attention, discussed in this article. We also summarize specific and sensitive methods, required for detection of *Pneumocystis* invasion and for distinguish colonization from the disease.

*Pneumocystis* spp*.* are unicellular, eukaryotic organisms occurring in lungs of many mammals. Five species-specific *Pneumocystis* species have been identified: *Pneumocystis carinii* and *Pneumocystis wakefieldiae* in rats, *Pneumocystis murina* in mice, *Pneumocystis oryctolagi* in rabbits, and *Pneumocystis jirovecii* in humans (Aliouat-Denis et al. 2008). *P. jirovecii* is a causative agent of *Pneumocystis* pneumonia (PcP; pneumocystosis), a particularly hazardous disease in case of people with impaired immune system. In immunocompetent people, infection caused by this pathogen may also lead to an asymptomatic carriage, which is an undesirable phenomenon, due to its contribution to *Pneumocystis* dissemination in population.

## Brief history

*Pneumocystis,* initially considered to be a protozoan, later has been assigned to the kingdom of fungi, due to its high genetic sequence homology with these organisms, as demonstrated by molecular studies (Edman et al*.*^1988^). Nevertheless, despite many similarities, *Pneumocystis* is an atypical fungus which differs in several respects from its relatives. One of such distinctive features, among others, is the presence of cholesterol in the *Pneumocystis* cell membrane, instead of ergosterol, which is the target of amphotericin B and ketoconazols. Therefore these drugs, commonly used as therapeutics in infections caused by other fungi, are ineffective in treatment of symptoms triggered by *P. jirovecii* (Kaneshiro et al. 1994).

Further DNA analyses have revealed that *Pneumocystis* species infecting lungs of various mammalian species are quite different and their infection is host specific—for example, individuals taken from rats and transferred to mice will not proliferate nor cause any symptoms of infection, while transmission to another rat will cause severe disease (Aliouat et al. 1994). After this discovery, *P. jirovecii,* formerly *Pneumocystis* f. sp. *hominis,* has been identified as a separate subspecies characteristic for humans. The name was given in honor of the Czech parasitologist Otto Jirovec, due to his important contribution in describing this organism in humans (Frenkel 1999).

## Structure and life cycle

*Pneumocystis* spp*.* has a biphasic life cycle with two distinct morphological forms: haploid trophozoites, constituting the proliferative stages, being asexual phase of the lifecycle, and cysts, representing a reproductive stage. Cysts are generated during the sexual phase, as a result of conjugation of trophozoites (Limper and Thomas 2007). Trophic forms predominate in lungs during the infection, while cysts have the major role in *Pneumocystis* propagation (Dumoulin et al. 2000).

## *Pneumocystis* pneumonia

Transmission of *P. jirovecii* cysts takes place through the airborne route, and usually, its presence in lungs is asymptomatic. However, people with impaired immunity, especially those with CD4+ T cell count below 200/μl (Phair et al. 1990), are still at risk of the development of *Pneumocystis* pneumonia due to *P. jirovecii* invasion. Symptoms induced by this disease are not specific: progressive dyspnoea, non-productive cough, low-grade fever, arterial partial pressure of oxygen below 65 mmHg, and chest radiographs demonstrating bilateral, interstitial shadowing (Barry and Johnson 2001). In computed tomography (CT) examination, the initial appearance of PcP is characterized by ground glass opacities, which are considered to disappear after introducing adequate therapy. Therefore, CT observations may also be useful while monitoring the effectiveness of treatment (Vogel et al. 2012). Moreover, it has been shown that the early stages of *P. jirovecii* proliferation induce alveolar macrophages activation and an increase of proinflammatory interleukins level, as well as changes in pulmonary surfactant. These anatomical and physiological changes may occur even during infection characterized by low fungal burden (Limper et al. 1989).

First cases of *Pneumocystis* pneumonia were observed in the 1960s, as an ailment caused by the presence of opportunistic pathogen in children with congenital T cell immunodeficiency, as well as in patients with hematological neoplasm (Walzer et al. 1976). The incidence of this disease markedly decreased after introducing anti-*Pneumocystis* chemoprophylaxis, but it has increased again after emergence of human immunodeficiency virus (HIV) in 1980s. PcP has been one of the main causes of morbidity and mortality among HIV-infected people and has been the most common AIDS-defining opportunistic infection in USA, developing in more than 60 % of patients during their disease course (Selik et al. 1987). PcP among those patients was characterized by a significant level of *Pneumocystis* proliferation, whereas the inflammatory reaction was weak. The PcP incidence has decreased again after introducing highly active anti-retrovirus therapy (HAART) and routine use of anti-PcP prophylaxis, as well as due to improved awareness of this infection among medical care workers (Kaplan et al. 2000). This decline demonstrates substantial restoration of the immune system due to HAART, which raises the CD4+ T cell level and thereby protects patients against opportunistic infections. PcP, the most common of such complications, still occurs primarily in patients deprived of medical care or unaware of their HIV status (Kaplan et al. 2000; Stringer 2002). There are also cases of PcP among patients already receiving prophylaxis, probably due to low adherence, poor performance at a very low CD4+ T cell counts or infection with resistant HIV strains (Kaplan et al. 2000).

**Fig. 1:**
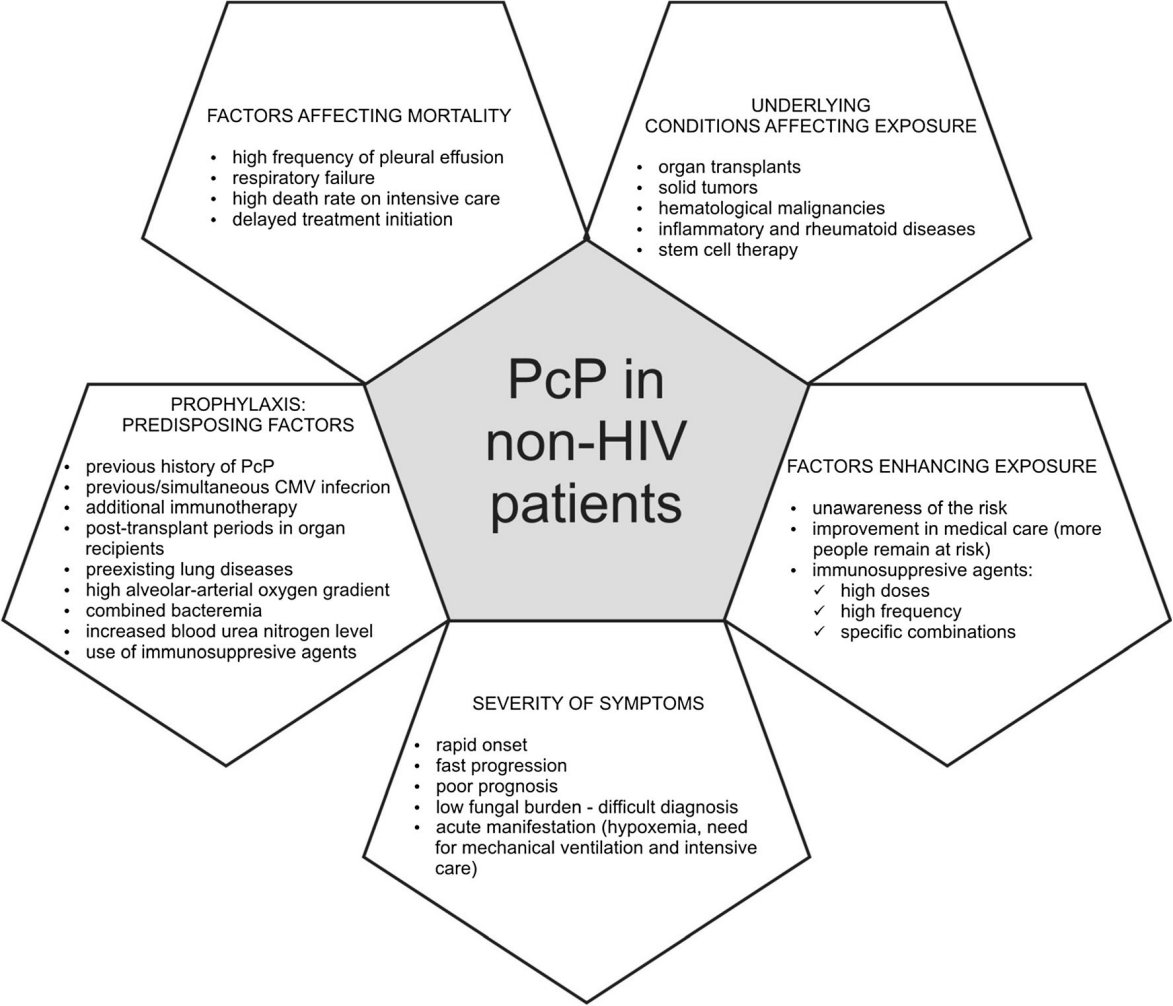
Major issues related to PcP in non-HIV patients

Another group of patients which are still at high risk of infection are non-HIV individuals with immunodeficiency caused by receiving cytotoxic and immunosupressive therapy for solid tumors, hematological malignancies or inflammatory and rheumatic diseases, as well as organ transplant recipients (Li et al. 2014). Increased number of cases of PcP in this group may be related to greater frequency of applications of immunosuppressants, usage of their higher doses, or to different combination of immunsuppressive agents which lead to enhanced susceptibility of patient to *Pneumocystis* infection (Ward and Donald 1999). In addition, due to improvements in medical care, more people with immune deficiency survive, remaining at risk of PcP. On the other hand, non-HIV patients are less aware that they are at risk of PcP, which often contributes to prolonged time from the onset of symptoms to treatment initiation. Thus, this group of patients represents a novel, serious public health care problem (Fig. 1).

## Non-HIV patients—the group of highest risk

It has been shown that PcP in non-HIV patients is characterized by more rapid onset and faster progression of symptoms, which are also more serious than in HIV-infected individuals. These symptoms include severe hypoxemia as well as greater need for intensive care and mechanical ventilation, which are often associated with poorer prognosis (Roblot et al. 2002). Therefore, death rates in this group of patients are significantly higher than in HIV-infected people, who present, in contrast, subacute disease course (Roux et al. 2014). The reason of this difference may be related to underlying condition: besides mortality, also proportion of cases requiring mechanical ventilation is higher in non-HIV than HIV-positive patients. Greater neutrophil count observed in non-HIV patients’ bronchoalveolar lavage fluids suggests that lung injuries caused by PcP are much more severe in these cases than in HIV patients (Monnet et al. 2008).

The most significant risk factor for PcP development in non-HIV immunosuppressed patients is the reduction of CD4+ T cell level, caused by the action of immunosuppressive agents, particularly cyclosporine A, antithymocyte globulin, as well as anti-rejection therapy for organ transplant recipients with high doses of glucocorticosteroids. Their effect disrupt host defense against *P. jirovecii*, which involves series of interactions between CD4+ T lymphocytes and polymorphonuclear cells, macrophages and proinflammatory mediators released by these cells. Therefore, depletion of CD4+ T cells inhibits proper reaction of immunity system (De Boer et al. 2011).

The study of Kofteridis et al. (2014), aimed to explore predisposing factors and clinical picture in non-HIV-infected people, has revealed that among this group of patients, the clinical parameters of PcP did not depend on the type of administered immunosuppression, although the risk of developing PcP is raised after rituximab therapy (Dęborska-Materkowska et al. 2014) or when the therapy consists of steroids together with cytotoxic agents. However, some data suggest that even malignancy itself can increase the PcP onset probability (Stapleton et al. 2015). Moreover, this paper demonstrated high frequency of pleural effusion in non-HIV patients with PcP, especially those with malignancies. Also, the mortality rate was the highest among individuals with solid tumors (Kofteridis et al. 2014). Though the death rate is associated with underlying diseases, the major factor influencing the prognosis of PcP is the development of respiratory failure. As compared with people infected with HIV, the death rates on intensive care unit for non-HIV patients are higher, as well as the necessity of implementing first-line non-invasive mechanical ventilation, which fails in a large proportion of these individuals (Monnet et al. 2008). Importantly, it is not the respiratory distress itself that causes death but the ensuing multi-organ failure (Stapleton et al. 2015).

Similar analyses performed by Kim et al. (2014) have demonstrated that in-hospital mortality was associated with factors such as high alveolar-arterial oxygen gradient, increased blood urea nitrogen level, preexisting lung disease, as well as combined bacteremia, which is also assumed to be an indirect marker of the severity of immunosuppressive status and susceptibility to PcP. Higher mortality rate has also been observed in patients requiring mechanical ventilation (Ko et al. 2014).

Another risk factor for PcP is previous or simultaneous cytomegalovirus (CMV) infection. This virus suppresses helper T and antigen presenting cells’ functions, thereby altering host immune response. Therefore, diagnosis of one opportunistic infection in susceptible patient, such as transplant recipient, suggests the possibility of existence of another one. Re-introduction of PcP prophylaxis is suggested whenever a patient is recognized with CMV infection or treated with additional immunosuppressive therapy for acute rejection episode after transplantation, due to heavier burden of immunosuppression in such cases. Moreover, prolonged prophylaxis is advisable for patients with a previous history of PcP (Borstnar et al. 2013).

## Transmission and colonization

Since the antibodies against *P. jirovecii* were found in majority of children under the age of 4 (Piffer et al. 1978), it has been initially assumed that PcP development is mostly derived from latent *Pneumocystis* infection, acquired in early life. The subsequent genotypic analysis have shown, however, that the primary infection may be cleaned in immunocompetent hosts, and the PcP is caused by re-infection with different strains, typical for place of residence rather than place of birth (Keely et al. 1995).

The transmission of *Pneumocystis* occurs via an airborne route. It has been demonstrated that immunocompetent hosts could serve as a reservoir of the fungus in population through transferring it from one individual to another when they are within sufficiently close distance, without causing symptomatic disease, until the pathogen reaches the immunocompromised host, in which PcP may develop (Gigliotti et al. 2003). Data obtained from experiments on mice and rats indicate that close-contact period as short as 1 day is enough to transmit infection from donors to immunocompetent carriers, which can carry low numbers of pathogen for a relatively long time, thereby contributing to *Pneumocystis* circulation in the population (Dumoulin et al. 2000). It is noteworthy that asymptomatic carriage is a transient event, rather than lifelong latency. This inference seems to be supported by the observation of the pathogen presence in HIV-positive patients after PcP for no longer than 9.5 months (Wakefield et al. 2003).

Asymptomatic carriage of *Pneumocystis* is a phenomenon known as colonization and is important for several reasons. Besides transmission of the pathogen to other people, colonized individuals are also at risk of PcP development in case of decreased immunity. Furthermore, colonization in individuals receiving anti-*Pneumocystis* prophylaxis for a long time may lead to selection of drug-resistant strains. Finally, even minor amounts of pathogen present in lungs may provoke host inflammatory response, which in turn leads to lung damage and plays a role in progression of lung disorders, such as chronic obstructive pulmonary disease or lung cancer (Probst et al. 2000). Colonization has been recognized in non-HIV immunosuppressed patients, who are particularly prone to become carriers, as well as in people with more subtle immunodeficiency, such as pregnant women or people with lung diseases (Vargas et al. 2003). Thus, pulmonary disorders may be both predisposing factors for *Pneumocystis* colonization, as well as conditions resulting from pathogen carriage. Taking into account that people with lung tissue defects are sputum producers, their colonization may be particularly risky due to their potentially enhanced ability to infection dissemination (Rivero et al. 2008).

Another issue associated with colonization is the difficulty in differentiating it from PcP during diagnosis. Detection methods utilized in most laboratories involve molecular techniques, but the standard cutoff value to distinguish colonization from active pneumocystosis has not been established yet. Therefore, when colonized patient experiences pneumonia with a different etiology, positive results of *Pneumocystis* detection may be misleading (Tasaka et al. 2014).

## Primary *Pneumocystis* infection in infants

As it was mentioned above, *Pneumocystis* infection/colonization occurs frequently among healthy children. Their common exposure to primary infection is suggested by the increased level of antibodies directed against *Pneumocystis* recorded during the first years of life. Yet, before they develop the properly functioning immune system for total elimination of the pathogen, they may transmit it to other susceptible hosts (Vargas et al. 2001). Molecular typing studies have demonstrated that identical fungal genotypes were harbored by children and adults with PcP, living in the same area (Totet et al. 2004). This finding is clearly showing the role of colonized infants in pathogen transmission. Additionally, the colonization in children appears to be frequent phenomenon during upper respiratory infections (Spencer et al. 2009).

Recent studies indicate that *P. jirovecii* infection in children occurs most frequently in the age range from 2 to 5 months, which overlaps with the most common age at which the increase in respiratory morbidity is observed (Vargas et al. 2013). In nasopharyngeal aspirates, taken from infants below 2 years of age presenting with mild respiratory symptoms, *P. jirovecii* DNA was detected in one third of cases. Positive samples were detected for the significantly younger age, yet, no specific symptoms of infection were perceived (Djawe et al. 2010a). It is presumed that colonization in infants may be associated with such disorders as bronchiolitis or even sudden unexpected infant death (SUID). Despite the fact that the results of autopsies performed on children have not demonstrated any significant difference in *Pneumocystis* prevalence between unexpected and explained death, its high incidence among SUID victims suggests that this pathogen still may be necessary, but not the sole, causative agent of the children unexpected death phenomenon (Vargas et al. 2007).

## Genetic diversity

Drug resistance of some *P. jirovecii* strains, responsible for failure of prophylaxis and/or treatment of infection, results often from the polymorphisms of different fungal genes (Kazanjian et al. 1998). Such genetic changes may also influence the epidemiological profiles of *P. jirovecii*, like pathogenicity or modes of transmission, as well as clinical outcome of PcP cases (Esteves et al. 2008). It has been shown that specific single-nucleotide polymorphisms (SNPs) in *Pneumocystis* genes were correlated with specific parameters of infection, such as fungal burden or type of progression and outcome of the disease (Esteves et al. 2011). Thus, molecular methods seem to be a perfect tool to study the genetic diversity among *P. jirovecii* individuals. In the future, analysis of the correlation between these different genotypes and a specific clinical picture caused by their invasion may be used to predict the PcP development, as well as to facilitate the decision about treatment and prophylaxis.

Genetic diversity of *P. jirovecii* strains is principally caused by gene variations resulting from SNPs presence. Several polymorphisms are regarded as highly informative markers for comparative analyses between the given genotype and a specific clinical or epidemiological data. For instance, it has been reported that SNP at the 85 position of the *P. jirovecii* mitochondrial large-subunit rRNA (*mtLSU rRNA*) gene, involved in basic mechanisms during translation, is useful to detect intraspecific differences between populations (Beard et al. 2000), whereas mutations in dihydropteroate synthase (*DHPS*) gene, presumably resulting from previous exposure to sulfa drug prophylaxis, have been linked to drug resistance and failure of anti-PcP treatment (Bento et al. 2013). In turn, data obtained during studies on mutations in the gene encoding DHFR enzyme from folic acid pathway demonstrate that five out of the six mutations conserved among unrelated species are located within the presumed active site of the enzyme. This observation allows the inference that SNPs distribution in this gene is not totally random, and they may have emerged by the exerted selective pressure of DHFR inhibitor, utilized in anti-*Pneumocystis* therapy. The outcome of this pressure is the acquisition of drug resistance, which can be observed as the failure of prophylaxis in patients infected with such strains. This effect seems to occur in response to mutations causing modifications in the enzyme’s structure, thereby diminishing its affinity for inhibitors (Nahimana et al. 2004).

Multilocus sequence typing (MLST) is considered to be a gold standard for analysis of genetic diversity, as it offers many advantages: reproducibility, possibility of exchanging data from different laboratories, as well as observations of polymorphisms at many loci together, at the same time. Maitte et al. (2013) have proposed the reduced MLST scheme, utilizing the three-locus analysis: *SOD* (superoxide dismutase), *CYB* (cytochrome B), and *mtLSU rRNA*. This scheme is more economical and easier to perform, at the same time preserving the sufficient discriminatory power. Thus, it is proposed to be used in many laboratories as an alternative strategy for preliminary investigations of PcP outbreaks. It can also detect infections of single patient by two, or even more, *P. jirovecii* isolates, which has been reported to be a common event.

Determination of the genotype by following the analysis of SNPs at single locus is much less sensitive and precise comparing with MLST. Multilocus analysis not only provides more comprehensive information, it can also be used to investigate epidemiological diversity. For instance, it has been used to define the epidemiological variability, suggesting differences in genotype frequencies between Old and New World *P. jirovecii* isolates caused by geographical component (Monroy-Vaca et al. 2014). The epidemiological factors, like geographical and climatic characteristics, may also have an impact on the circulation of specific *P. jirovecii* genotypes within designated areas (Esteves et al. 2008; Dimonte et al. 2013). The geographical variation in the prevalence of gene mutations associated with drug resistance may be caused by differences in the types of PcP prophylaxis used in various regions (Esteves et al. 2008). Such epidemiological factors are yet another aspect of risk connected with colonization and transmission of different *P. jirovecii* strains (Beard et al. 2000).

## Detection of *P. jirovecii* and PcP diagnosis

The rapid diagnosis of PcP is crucial especially among non-HIV patients due to the severe symptoms, which could be avoided by early enough implementation of treatment, since it has been shown that acceleration of treatment initiation by only one day can be associated with significantly reduced mortality (Roux et al. 2014). However, low fungal burden, specific for samples taken from non-HIV patients with PcP, often hinders the detection of infection and may lead to false-negative results. It is particularly difficult to set the right diagnosis in such case using solely the gold standard – microscopic visualization of *Pneumocystis*, which additionally depends on skills and experience of the observer (Limper et al. 1989). Furthermore, another feature differentiating *Pneumocystis* species from other fungi is the lack of appropriate systems for *in vitro* culturing, which hinders the research on this organism even more (Stringer 2002). Therefore, diagnostic methods based on both microscopic observations and genotyping are involved.

Conventional staining methods can be used for microscopic observation of *P. jirovecii*, as well as indirect immunofluorescence staining with monoclonal antibodies directed against *P. jirovecii* cysts, which is faster and unambiguous. However, such visualizations have some limitations. First of all, they are conducted on specimens collected during sputum induction which may not be tolerated by some patients, or bronchoalveolar lavage (BAL) specimens and lung biopsies, which are invasive methods. In addition to that, histological detection relies mainly on the experience of the observer and is time-consuming. Finally, low fungal burden, impeding the microscopic observation especially in non-HIV patients’ cases, may lead to false-negative diagnosis (Limper et al. 1989).

Therefore, detection of *Pneumocystis* DNA whereby PCR assays amplifying various genetic fragments has been implicated. The sensitivity of diagnosis can be improved either due to application of multicopy gene target, like *mtLSU rRNA* or *msg*, or by using nested PCR that consists of two rounds of amplification, thereby increasing detection rate (Alvarez-Martínez et al. 2006). Introduction of PCR method has revealed the new population of people with detectable *Pneumocystis* DNA, even though just few or no organisms are visualized (Peterson and Cushion 2005). Widespread use of conventional PCR method in clinical practice has one limitation, namely insufficient specificity and lack of quantification, required for distinguishing between PcP and colonization. The improved version of the PCR assay has emerged, which possess those advantageous features – quantitative real-time PCR (qRT-PCR). It is also beneficial over the nested PCR, due to limitation of the risk of DNA carry-over and reduction of time consumption, as it is a one-step process (Alanio et al. 2011). However, interpretation of the results is still ambiguous and standard quantities to set the right diagnosis and differentiate PcP from colonization have not been determined yet. Besides, the remaining impediment is the invasiveness of BAL performance, which is impossible in patients with limited respiratory function. The solution in such cases is the utilization of minimally invasive, readily available and not expensive samples, the conditions met by the detection of serological markers of PcP in blood specimens.

One of such markers is the component present in cell walls of many fungi, including *P. jirovecii* cysts—(1 → 3)-B-d-glucan (BG). It has been shown that its level is significantly higher in serum of patients with definite or probable PcP in comparison with the colonized people, therefore positive results of BG measurements may be indicative of the requirement for anti-PcP treatment (Tasaka et al. 2014). Even though this assay is not specific for *Pneumocystis,* it may serve as an additional confirming tool in situations when detection by PCR does not give clear results. All available elements—CD4+ T cell counts, symptoms, and CT findings—have still to be considered during making clinical decision and BG levels alone cannot be the proof of PcP, because of the non-specificity of this test. However, its negative predictive value (99.8 %) can actually exclude PcP which is suitable in patients who cannot undergo bronchoscopy or in those with low clinical suspicion of PcP (Held et al. 2010). The analyses have demonstrated that positive BG and PCR results allowed applying an anti-PcP treatment in the microscopically negative patients, thus increasing the survival rate (Matsumura et al. 2014).

It has been deduced that, in complementation to PCR, BG measurements are significant for discrimination between PcP and colonization (Esteves et al. 2014). Different values for detection have been proposed: Salerno et al. (2014) suggested the cutoff of 300 pg ml^−1^ for the diagnosis in AIDS-positive patients, whereas Tasaka et al. (2014) estimated the cutoff level for discrimination of 33.5 pg ml^−1^. In turn, Esteves et al. (2014) have shown that the highest level of median BG was observed in samples taken from PcP patients (270 pg ml^−1^), followed by colonized people with almost four times lower results (67 pg ml^−1^), while patients with other pulmonary diseases had the lowest BG level (36 pg ml^−1^), almost equal to that of healthy people (31 pg ml^−1^).

Moreover, it has been observed that lactate dehydrogenase (LDH) serum levels are also increased in PcP patients samples. This enzyme is released from host cells in response to their cytoplasmic membrane damage, in this case probably caused by lung damage following the pathogen presence (Esteves et al. 2014). In healthy people, LDH levels are within the range of 100–350 U l^−1^. LDH levels cannot, however, be used to distinguish between *P. jirovecii* colonization and PcP, because they are too similar within the samples of these two groups of patients (Esteves et al. 2014). After all, BG and LDH levels are positively correlated; therefore, they can be used together as an adjunctive reliability factor during setting diagnosis concerning PcP (Esteves et al. 2014; Borstnar et al. 2013).

Another non-invasive approach to diagnose PcP is the utilization of enzyme-linked immunosorbent assay (ELISA). One of the relevant target proteins is the major surface glycoprotein (Msg) of *Pneumocystis,* the protein involved in interactions with many host molecules and in attachment to alveolar epithelial cells (Stringer and Keely 2001). The carboxy-terminal region of this protein is relatively conserved, and antibodies directed to this fragment have been shown to be at higher level in samples taken from HIV patients with active PcP than from patients with pneumonia caused by other factors (Djawe et al. 2010b). Another important target protein for immunochemical detection is the *Pneumocystis* protease, kexin (Kex), encoded by a single-copy gene (Kutty and Kovacs 2003). It has been shown that even low quantities of Kex antibody level were correlated with subsequent episodes of PcP, suggesting that it may be another, early marker of future PcP risk in HIV-positive individuals (Gingo et al. 2011).

## Treatment

As it was mentioned before, *Pneumocystis* infection cannot be treated with typical drugs utilized in fungal infections. Instead, the mechanism of disrupting folic acid pathway in *Pneumocystis* organisms is a good target for therapeutic agents—such therapy relies on the fact that *P. jirovecii* can not acquire folic acid from the environment, making it utterly dependent on *de novo* synthesis. Folic acid is required for synthesis of purines, glycine, and thymidylate—necessary for proper organism functioning. Metabolic pathway of this synthesis involves two enzymes important in anti-PcP prophylaxis—dihydropteroate synthase (DHPS) and dihydrofolate reductase (DHFR) (Volpe et al. 1993). Such prophylaxis consists of the combination of trimethoprim (TMP; an inhibitor of DHFR) and a sulfa drug, sulphamethoxazole (SMX; an inhibitor of DHPS) (Lobo et al. 2013)*.* The inhibition of folic acid synthesis leads to the inability of production of proteins due to the lack of amino acids, as well as to impossibility of DNA synthesis and repair, because of nucleotides deficiency.

Studies performed on pathogens isolated form patients who failed sulfa drugs prophylaxis have demonstrated that characteristic non-synonymous mutations occur frequently in *DHPS* gene, leading to alterations in the enzyme’s structure and hence reduced affinity to sulfa drugs (Armstrong et al. 2000). Such resistance occurs as a result of sulfa drug treatment employment in many other disorders, which triggers the positive selective pressure on the DHPS gene in *Pneumocystis* strains (Kazanjian et al. 1998). Anyhow, TMP-SMX is the most frequently used anti-*Pneumocystis* therapy. Moreover, it has been shown that the addition of low doses of caspofungin (caspofungin acetate, active against cysts) which acts as an inhibitor of the (1 → 3)-B-d-glucan synthase, to TMP-SMX (active against cysts and trophic forms) may enhance the inhibition effect (Lobo et al. 2013). The therapy consisting of the TMP-SMX/caspofungin provides fast action, and it is more effective than with utilizing these drugs alone, due to the simultaneous fungistatic and fungicidal effect, both on cysts and trophic forms of pathogen. Moreover, this drug combination may reduce harmful inflammatory response induced by BG (Lobo et al. 2013).

## Prevention of PcP

Although PcP prophylaxis for susceptible, immunocompromised patients has become the standard of care, the optimal scheme and duration of therapy have not been defined. In case of organ recipients, the early post-transplant period is the considered highest risk time for infection, albeit there is a growing evidence of late PcP recognition, even above one year after transplantation (Wu et al. 2012). In turn, in HIV-infected patients whose CD4+ T lymphocyte counts fall below critical level or in those with previous episodes of PcP, prophylaxis is advisable and it should be continued until CD4+ T cell level rises again above 200/μl, due to HAART therapy (From the Centers for Disease Control 1992).

Nonetheless, it has been confirmed that clinical centers undertake to perform PcP prophylaxis, among others in children with cancers (Caselli et al. 2014), resulting in effective protection of patients. Non-adherence to this prophylaxis remains the only reason for its failure; therefore, a simpler regimen with a shorter duration may be expected to improve the effectiveness. It has been suggested that a single-day prophylactic scheme with TMP-SMX may be efficient in preventing PcP in children with solid tumor, leukemia, or lymphoma, treated with intensive chemotherapy. This strategy may be also of potential application in other patient populations with immunosuppression (Caselli et al. 2014).

## Conclusions

Due to the development of our knowledge about complications associated with HIV infection, as well as subsequent introduction of HAART therapy, it has been possible to reduce the mortality and morbidity caused by PcP among patients infected with this virus. However, despite the utilization of anti-*Pneumocystis* prophylaxis, this disease is still a serious public health problem, affecting mainly non-HIV patients being under immunosupression. In addition to that, many healthy, immunocompetent people are colonized with this fungus without any symptoms, therefore without the awareness of the possibility of participating in pathogen transmission to susceptible hosts. Apparently, the majority of infants are also members of colonized population. Hence, *P. jirovecii* carriage is assumed to contribute to some respiratory disorders. From all the evidence given above, it is clear that sensitive methods for detection of this fungus, as well as for distinguishing PcP from asymptomatic colonization, are essential in order to increase the survival rate of vulnerable patients. The lack of appropriate system for culturing *P. jirovecii* is the major impediment in research enabling expanding our knowledge about this organism, which can be instead bypassed by more specific molecular studies, including research on *Pneumocystis* epidemiology.

## Abbreviations

PcP: *Pneumocystis* pneumonia
DHFR: Dihydrofolate reductase
DHPS: Dihydropteroate synthase
HAART: Highly active anti-retrovirus therapy
SNP: Single-nucleotide polymorphism
MLST: Multilocus sequence typing
BG: (1 → 3)-B-d-glucan
LDH: Lactate dehydrogenase
TMP-SMX: Trimethoprim-sulphamethoxazole
